# Coding-Complete Genome Sequences of a Delta Subvariant (AY.33) of SARS-CoV-2 Obtained from Moroccan COVID-19 Patients

**DOI:** 10.1128/mra.01099-21

**Published:** 2022-02-03

**Authors:** Mouhssine Hemlali, Taha Chouati, Hamza Ghammaz, Marouane Melloul, Sanaâ Alaoui Amine, Safaa Rhoulam, Nadia Touil, Khalid Ennibi, Hicham Oumzil, Rhajaoui Mohamed, Aguenaou Hassan, Elmostafa El Fahime

**Affiliations:** a Molecular Biology and Functional Genomics Platform, National Center for Scientific and Technical Research (CNRST), Rabat, Morocco; b Neuroscience and Neurogenetics Research Team, Faculty of Medicine and Pharmacy, University Mohammed V, Rabat, Morocco; c Cell Culture Unit, Center of Virology, Infectious, and Tropical Diseases, Med V Military Hospital, Rabat, Morocco; d Microbiology and Molecular Biology Team, Center of Plant and Microbial Biotechnology, Biodiversity, and Environment, Faculty of Sciences, Mohammed V University of Rabat, Rabat, Morocco; e Ibn Tofaïl University-CNESTEN, Joint Research Unit in Nutrition and Food, RDC-Nutrition AFRA/IAEA, Rabat-Kénitra, Morocco; f National Institute of Hygiene, Rabat, Morocco; g National Influenza Center, Virology Department, Institut National d’Hygiène, Rabat, Morocco; h Virology and Infectious Tropical Diseases Center, Med V Military Hospital, Rabat, Morocco; i Genomic Center for Human Pathologies (GENOPATH), Faculty of Medicine and Pharmacy, University Mohammed V, Rabat, Morocco; DOE Joint Genome Institute

## Abstract

We report here the complete genome sequences of severe acute respiratory syndrome coronavirus 2 (SARS-CoV-2) strains obtained from Moroccan patients with COVID-19. The analysis of these sequences indicates that the identified strains belong to the AY.33 sublineage of the Delta variant.

## ANNOUNCEMENT

In December 2019, the severe acute respiratory syndrome coronavirus 2 (SARS-CoV-2), first described in the city of Wuhan in the Republic of China, and was identified as the etiological agent of the coronavirus disease 19 (COVID-19) (1). SARS-CoV-2 is a member of the *Coronaviridae* family and *Betacoronavirus* genus (2). In Morocco, as of 3 October 2021, more than 935,000 COVID cases had been reported, with a comparatively high death rate (14,339 deaths) (www.covidmaroc.ma). Here, we report the complete genome sequences of two SARS-CoV-2 strains recovered from Moroccan patients.

As part of the genomic surveillance against SARS-CoV-2, nasopharyngeal swabs from two Moroccan patients living in Casablanca were received on 7 and 8 July 2021, respectively. The viral RNA was extracted using the MagPurix instrument (Zinexts Life Science, Taiwan). Confirmation of the infection of the patients with SARS-CoV-2 was done by reverse transcription-quantitative PCR (qRT-PCR) using the GeneFinder COVID-19 Plus RealAmp kit at the National Center for Scientific and Technical Research, Rabat, Morocco. The quantity and quality of RNA were measured with a Qubit 4 fluorometer (Invitrogen, Thermo Fisher Scientific, USA) using the Qubit RNA high-sensitivity (HS) assay kit (Invitrogen, Thermo Fisher Scientific).

The viral RNA was reverse-transcribed using a SuperScript VILO cDNA synthesis kit (Invitrogen, Thermo Fisher Scientific, USA) and processed for library preparation using the Ion AmpliSeq SARS-CoV-2 research panel (Thermo Fisher, USA). The DNA libraries were then used for template preparation and chip loading on the Ion Chef instrument. Sequencing was carried out on the Ion S5 sequencer. Then, using Unipro UGENE version 38, a consensus sequence was generated by mapping the reads against the Wuhan-Hu-1 reference sequence (GenBank accession number NC_045512.2). All tools were run with default parameters unless otherwise specified.

The genomes for samples 5858 and 5900 were obtained with an average read depth of 3,675× and 5,445× and showed lengths of 29,818 bp and 29,822 bp, respectively, and a GC content of 37.9%. The quality of the sequences was monitored using the Nextclade web tool version 1.7.2 (https://clades.nextstrain.org), although the determination of the mutations was carried out using the web application Cov-GLUE (http://cov-glue.cvr.gla.ac.uk/), and the lineage and sublineage were obtained using the web application Pangolin (https://pangolin.cog-uk.io/).

**FIG 1:**
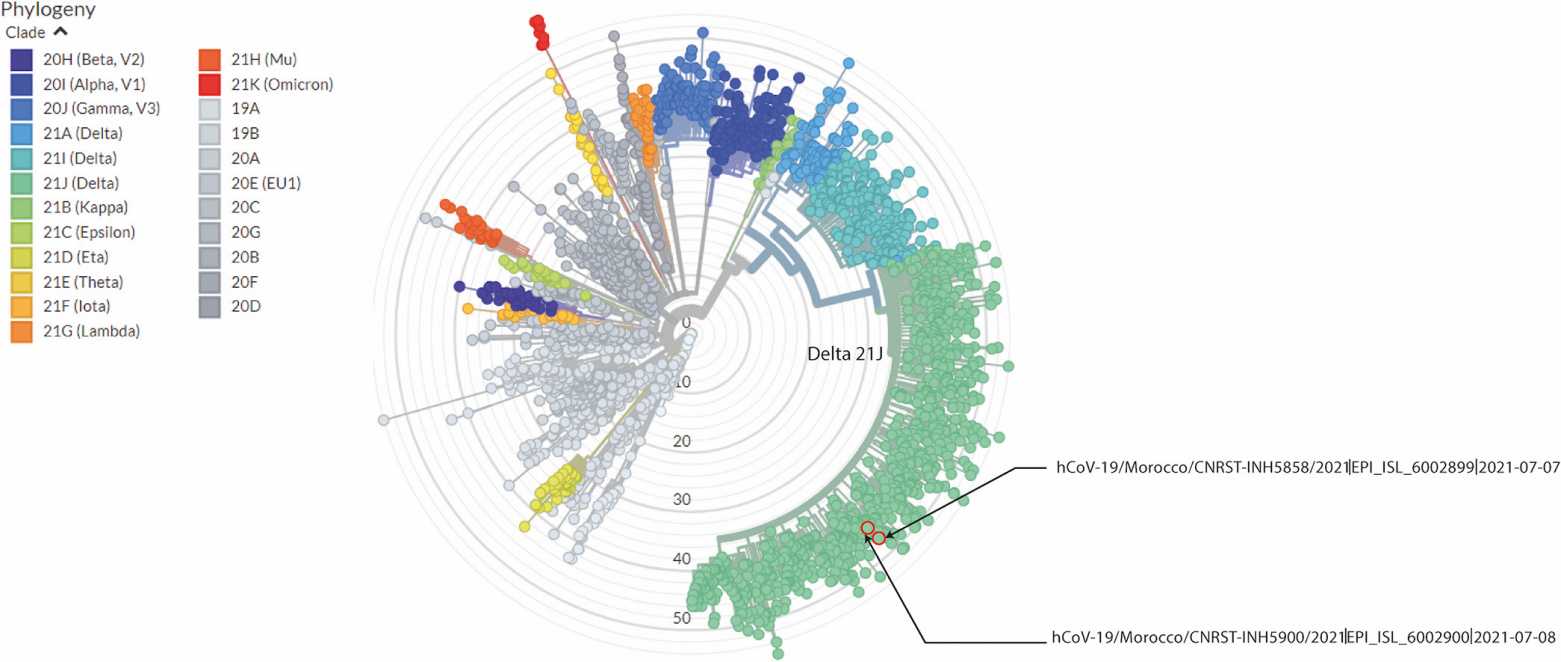
Phylogenetic tree of SARS-CoV-2 generated from Nextstrain using the Nextclade maximum likelihood module. The hCoV-19/5858 and hCoV-19/5900 strains are situated in the Delta lineage (green) and, more specifically, in the AY.33 sublineage.

Both samples belonged to clade GK, sublineage AY.33 of Delta variant B1.617.2 (Delta 2). The mutations within the viral genome of sample 5858 (31 mutations) and sample 5900 (30 mutations) are summarized in Table 1, and their localization within global SARS-CoV-2 diversity are illustrated in Fig. 1.

**TABLE 1:** Amino acid variants of the sequences of hCoV-2019/5858 and hCoV2019-5900 in comparison to the reference strain (GenBank accession number NC_045512.2) using the web application CoV-GLUE

Open reading frame	Variants for:	
	hCoV-2019/5858	hCoV-2019/5900
ORF 1a	A1306S P2046L P2287S V2930L T3255I L3606F T3646A I4049V	A1306S P2046L P2287S V2930L T3255I T3646A
ORF 1b	P314L G662S P1000L A1918V	P314L G662S P1000L A1918V
S	T19R T29A G142D R158G T250I T299I L452R T478K Q613H D614G P681R D950N E157-F158-	T19R T29A G142D R158G T250I T299I L452R T478K Q613H D614G P681R D950N E156-F157-
ORF 3a	S26L	S26L
M		I82T
ORF 7a	V82A T120I	V82A T120I
ORF 7b	T40I	T40I
ORF 8	D119- F120-	
ORF 9B	T60A	T60A
N	D63G R203M G215C N377Y	D63G R203M G215C N377Y

The genomes of both samples accumulated 4 new amino acid changes within the Spike protein (T29A, T50I, T299I, and Q613) in addition to the 10 modifications that characterize the Delta 2 variant.

T487K, which results in a significant modification in the conformation of the receptor binding domain (RBD), was linked to an increase in RBD binding affinity to the cell surface angiotensin converting enzyme 2 (ACE2) receptor (3). In addition, Q613H was also reported as an amino acid modification of little-known impact; however, given its close proximity to the well-known D614G, it is possible that this amino acid change would enhance the role of the latter in terms of virus transmissibility (4, 5).

Furthermore, among amino acid modifications affecting the receptor binding motif (RBM) region, L452R is present in variants which exhibit high binding affinity to the ACE2 receptor and may also contribute to humoral immune escape (6). Another interesting amino acid change is P681R, located between subunits S1 and S2. It has been established that the P681R change increases S1/S2 cleavage, which induces a quicker fusion between virus and cell membrane, thus facilitating viral transmissibility (7).

Finally, the N-terminal domain (NTD) (an essential binding site for antibody 4A8 [8]) is altered through G142D. This mutation contributes to changing the structure of the NTD, resulting in the binding prevention of neutralization antibodies (NAb) to this domain (9). The characteristic B.1.617.2 and B.1.617.3 deletions 157 to 158 can also be observed and constitute another mechanism by which the virus acquired vaccine escape abilities (10).

The emergence and spread of new variants derived from volatile organic compounds (VOCs) are a global concern, their escape from vaccination is not yet well studied, and the genomic surveillance of the genome of SARS-CoV-2 remains a primary approach to identify variants and prevent their spread.

### Data availability.

The SARS-CoV-2 genome sequences 5858 and 5900 were deposited into the GISAID database under the identifiers EPI_ISL_6002899 and EPI_ISL_6002900, respectively, and in NCBI GenBank under the accession numbers OL375239 and OL375240, respectively. Raw reads from next-generation sequencing (NGS) are available in the BioProject database under SRA number PRJNA780922. The specific raw read library identifiers of samples 5858 and 5900 are SRX13150847 and SRX13150848, respectively.
